# GametesOmics: A Comprehensive Multi-omics Database for Exploring the Gametogenesis in Humans and Mice

**DOI:** 10.1093/gpbjnl/qzad004

**Published:** 2023-12-22

**Authors:** Jianting An, Jing Wang, Siming Kong, Shi Song, Wei Chen, Peng Yuan, Qilong He, Yidong Chen, Ye Li, Yi Yang, Wei Wang, Rong Li, Liying Yan, Zhiqiang Yan, Jie Qiao

**Affiliations:** State Key Laboratory of Female Fertility Promotion, Center for Reproductive Medicine, Department of Obstetrics and Gynecology, Peking University Third Hospital, Beijing 100191, China; National Clinical Research Center for Obstetrics and Gynecology, Peking University Third Hospital, Beijing 100191, China; Key Laboratory of Assisted Reproduction, Ministry of Education, Peking University, Beijing 100191, China; Beijing Key Laboratory of Reproductive Endocrinology and Assisted Reproductive Technology, Beijing 100191, China; Peking-Tsinghua Center for Life Sciences, Peking University, Beijing 100871, China; State Key Laboratory of Female Fertility Promotion, Center for Reproductive Medicine, Department of Obstetrics and Gynecology, Peking University Third Hospital, Beijing 100191, China; National Clinical Research Center for Obstetrics and Gynecology, Peking University Third Hospital, Beijing 100191, China; Key Laboratory of Assisted Reproduction, Ministry of Education, Peking University, Beijing 100191, China; Beijing Key Laboratory of Reproductive Endocrinology and Assisted Reproductive Technology, Beijing 100191, China; State Key Laboratory of Female Fertility Promotion, Center for Reproductive Medicine, Department of Obstetrics and Gynecology, Peking University Third Hospital, Beijing 100191, China; National Clinical Research Center for Obstetrics and Gynecology, Peking University Third Hospital, Beijing 100191, China; Key Laboratory of Assisted Reproduction, Ministry of Education, Peking University, Beijing 100191, China; Beijing Key Laboratory of Reproductive Endocrinology and Assisted Reproductive Technology, Beijing 100191, China; Peking-Tsinghua Center for Life Sciences, Peking University, Beijing 100871, China; State Key Laboratory of Female Fertility Promotion, Center for Reproductive Medicine, Department of Obstetrics and Gynecology, Peking University Third Hospital, Beijing 100191, China; National Clinical Research Center for Obstetrics and Gynecology, Peking University Third Hospital, Beijing 100191, China; Key Laboratory of Assisted Reproduction, Ministry of Education, Peking University, Beijing 100191, China; Beijing Key Laboratory of Reproductive Endocrinology and Assisted Reproductive Technology, Beijing 100191, China; State Key Laboratory of Female Fertility Promotion, Center for Reproductive Medicine, Department of Obstetrics and Gynecology, Peking University Third Hospital, Beijing 100191, China; National Clinical Research Center for Obstetrics and Gynecology, Peking University Third Hospital, Beijing 100191, China; Key Laboratory of Assisted Reproduction, Ministry of Education, Peking University, Beijing 100191, China; Beijing Key Laboratory of Reproductive Endocrinology and Assisted Reproductive Technology, Beijing 100191, China; State Key Laboratory of Female Fertility Promotion, Center for Reproductive Medicine, Department of Obstetrics and Gynecology, Peking University Third Hospital, Beijing 100191, China; National Clinical Research Center for Obstetrics and Gynecology, Peking University Third Hospital, Beijing 100191, China; Key Laboratory of Assisted Reproduction, Ministry of Education, Peking University, Beijing 100191, China; Beijing Key Laboratory of Reproductive Endocrinology and Assisted Reproductive Technology, Beijing 100191, China; State Key Laboratory of Female Fertility Promotion, Center for Reproductive Medicine, Department of Obstetrics and Gynecology, Peking University Third Hospital, Beijing 100191, China; National Clinical Research Center for Obstetrics and Gynecology, Peking University Third Hospital, Beijing 100191, China; Key Laboratory of Assisted Reproduction, Ministry of Education, Peking University, Beijing 100191, China; Beijing Key Laboratory of Reproductive Endocrinology and Assisted Reproductive Technology, Beijing 100191, China; State Key Laboratory of Female Fertility Promotion, Center for Reproductive Medicine, Department of Obstetrics and Gynecology, Peking University Third Hospital, Beijing 100191, China; National Clinical Research Center for Obstetrics and Gynecology, Peking University Third Hospital, Beijing 100191, China; Key Laboratory of Assisted Reproduction, Ministry of Education, Peking University, Beijing 100191, China; Beijing Key Laboratory of Reproductive Endocrinology and Assisted Reproductive Technology, Beijing 100191, China; State Key Laboratory of Female Fertility Promotion, Center for Reproductive Medicine, Department of Obstetrics and Gynecology, Peking University Third Hospital, Beijing 100191, China; National Clinical Research Center for Obstetrics and Gynecology, Peking University Third Hospital, Beijing 100191, China; Key Laboratory of Assisted Reproduction, Ministry of Education, Peking University, Beijing 100191, China; Beijing Key Laboratory of Reproductive Endocrinology and Assisted Reproductive Technology, Beijing 100191, China; State Key Laboratory of Female Fertility Promotion, Center for Reproductive Medicine, Department of Obstetrics and Gynecology, Peking University Third Hospital, Beijing 100191, China; National Clinical Research Center for Obstetrics and Gynecology, Peking University Third Hospital, Beijing 100191, China; Key Laboratory of Assisted Reproduction, Ministry of Education, Peking University, Beijing 100191, China; Beijing Key Laboratory of Reproductive Endocrinology and Assisted Reproductive Technology, Beijing 100191, China; State Key Laboratory of Female Fertility Promotion, Center for Reproductive Medicine, Department of Obstetrics and Gynecology, Peking University Third Hospital, Beijing 100191, China; National Clinical Research Center for Obstetrics and Gynecology, Peking University Third Hospital, Beijing 100191, China; Key Laboratory of Assisted Reproduction, Ministry of Education, Peking University, Beijing 100191, China; Beijing Key Laboratory of Reproductive Endocrinology and Assisted Reproductive Technology, Beijing 100191, China; Peking-Tsinghua Center for Life Sciences, Peking University, Beijing 100871, China; State Key Laboratory of Female Fertility Promotion, Center for Reproductive Medicine, Department of Obstetrics and Gynecology, Peking University Third Hospital, Beijing 100191, China; National Clinical Research Center for Obstetrics and Gynecology, Peking University Third Hospital, Beijing 100191, China; Key Laboratory of Assisted Reproduction, Ministry of Education, Peking University, Beijing 100191, China; Beijing Key Laboratory of Reproductive Endocrinology and Assisted Reproductive Technology, Beijing 100191, China; State Key Laboratory of Female Fertility Promotion, Center for Reproductive Medicine, Department of Obstetrics and Gynecology, Peking University Third Hospital, Beijing 100191, China; National Clinical Research Center for Obstetrics and Gynecology, Peking University Third Hospital, Beijing 100191, China; Key Laboratory of Assisted Reproduction, Ministry of Education, Peking University, Beijing 100191, China; Beijing Key Laboratory of Reproductive Endocrinology and Assisted Reproductive Technology, Beijing 100191, China; State Key Laboratory of Female Fertility Promotion, Center for Reproductive Medicine, Department of Obstetrics and Gynecology, Peking University Third Hospital, Beijing 100191, China; National Clinical Research Center for Obstetrics and Gynecology, Peking University Third Hospital, Beijing 100191, China; Key Laboratory of Assisted Reproduction, Ministry of Education, Peking University, Beijing 100191, China; Beijing Key Laboratory of Reproductive Endocrinology and Assisted Reproductive Technology, Beijing 100191, China; State Key Laboratory of Female Fertility Promotion, Center for Reproductive Medicine, Department of Obstetrics and Gynecology, Peking University Third Hospital, Beijing 100191, China; National Clinical Research Center for Obstetrics and Gynecology, Peking University Third Hospital, Beijing 100191, China; Key Laboratory of Assisted Reproduction, Ministry of Education, Peking University, Beijing 100191, China; Beijing Key Laboratory of Reproductive Endocrinology and Assisted Reproductive Technology, Beijing 100191, China; Peking-Tsinghua Center for Life Sciences, Peking University, Beijing 100871, China; Beijing Advanced Innovation Center for Genomics, Beijing 100191, China

**Keywords:** Gametogenesis, Oogenesis, Spermatogenesis, Transcriptomics, Epigenomics

## Abstract

Gametogenesis plays an important role in the reproduction and evolution of species. The transcriptomic and epigenetic alterations in this process can influence the reproductive capacity, fertilization, and embryonic development. The rapidly increasing single-cell studies have provided valuable multi-omics resources. However, data from different layers and sequencing platforms have not been uniformed and integrated, which greatly limits their use for exploring the molecular mechanisms that underlie oogenesis and spermatogenesis. Here, we develop GametesOmics, a comprehensive database that integrates the data of gene expression, DNA methylation, and chromatin accessibility during oogenesis and spermatogenesis in humans and mice. GametesOmics provides a user-friendly website and various tools, including Search and Advanced Search for querying the expression and epigenetic modification(s) of each gene; Tools with Differentially expressed gene (DEG) analysis for identifying DEGs, Correlation analysis for demonstrating the genetic and epigenetic changes, Visualization for displaying single-cell clusters and screening marker genes as well as master transcription factors (TFs), and MethylView for studying the genomic distribution of epigenetic modifications. GametesOmics also provides Genome Browser and Ortholog for tracking and comparing gene expression, DNA methylation, and chromatin accessibility between humans and mice. GametesOmics offers a comprehensive resource for biologists and clinicians to decipher the cell fate transition in germ cell development, and can be accessed at http://gametesomics.cn/.

## Introduction

Gametogenesis is a key process in reproduction and evolution of species, during which diploid germ cells undergo mitotic and meiotic divisions, and then differentiate into mature haploid gametes including eggs (oogenesis) and sperms (spermatogenesis) [1,2]. The complex molecular regulatory mechanism involving genetic and epigenetic changes supports the successful genesis of the gametes [3–5]. Nowadays, the rapid development of single-cell sequencing technologies has broadened our understanding of this process and provided a large volume of omics data [6–10]. However, these data have not been integrated or normalized for utilization, which hampers biologists and clinicians to explore the molecular mechanisms that underlie oogenesis as well as spermatogenesis.

Several databases contain valuable omics data of humans and mice. Deeply Integrated Single-Cell Omics data (DISCO) database collects the single-cell RNA sequencing (RNA-seq) datasets covering various tissues and cell types in human, which provides a comprehensive platform for exploring gene expression across different cell types and human tissues [11]. scMethBank database includes DNA methylation data across several species including human and mouse, and provides many useful functions such as search, browsing, and visualization for researchers [12]. dbEmbryo and DevOmics integrate multi-omics sequencing data across different developmental stages in human and mouse early embryos [13,14]. EmAtlas exhibits the spatiotemporal landscape of human and mouse embryos from the perspective of cell, tissue, genome, gene, and protein levels [15]. These current databases greatly promote the understanding of the genetic and epigenetic mechanisms in the development of humans and mice. However, they may lack the collection of comprehensive multi-omics sequencing data of the gametogenesis and online tools for exploring specific stepwise process regarding the oogenesis and spermatogenesis, which may hinder the exploration into the landscape and regulatory mechanisms of the development process during oogenesis and spermatogenesis. Therefore, it is highly desirable to construct a customized database that includes multi-omics data across complete developmental stages and offers user-friendly tools for identifying key regulators of cell fate transition in gametogenesis.

With the aim of establishing a comprehensive unified atlas of oogenesis and spermatogenesis, we developed GametesOmics (http://gametesomics.cn/), a multi-omics database which integrates the data of gene expression, DNA methylation, and chromatin accessibility spanning non-growing oocyte, growing oocyte, fully-grown oocyte (FGO), metaphase I oocyte, and metaphase II oocyte during oogenesis, as well as spermatogonia stem cell, spermatogonia, spermatocyte, spermatid, and mature sperm during spermatogenesis, in humans and mice. It provides various functions for querying and displaying gene expression as well as epigenetic modification changes, performing differential analysis, visualizing single-cell clusters, identifying master transcription factors (TFs), and tracking homologous genes’ expression in humans and mice. GametesOmics helps researchers study the synergistic change between different layers and explore human and mouse homologous key factors that trigger the development of the gametes, so as to decode regulatory mechanisms in cell fate determination in gametogenesis.

## Database implementation

### Data collection

We retrieved studies in PubMed (https://pubmed.ncbi.nlm.nih.gov/), Gene Expression Omnibus (GEO, https://www.ncbi.nlm.nih.gov/geo/), Sequence Read Archive (SRA, https://www.ncbi.nlm.nih.gov/sra/), and European Nucleotide Archive (ENA, https://www.ebi.ac.uk/ena/browser/) using the arrangement and combination of the keywords including “gametogenesis”, “oogenesis”, “oocyte”, “spermatogonia”, “sperm”, “RNA-seq”, “transcription”, “methylation”, and “chromatin accessibility”, and obtained related studies containing the single-cell sequencing datasets of gametogenesis in humans and mice. These studies used appropriate number of samples to demonstrate their discoveries. In consideration of the data quality and comparability, we made efforts to choose the datasets that cover the most complete developmental stages, with the largest number of samples and the most advanced sequencing methods as well as the same sequencing platforms to ensure the data consistency as possible.

We collected 6689 samples generated by RNA-seq for gene expression (4381 samples), whole-genome bisulfite sequencing (BS-seq) / chromatin overall omic-scale landscape sequencing (COOL-seq) [WCG sites (including ACG or TCG)] for DNA methylation (1162 samples), and COOL-seq [GCH sites (including GCA, GCC, or GCT)] for chromatin accessibility (1146 samples) across oogenesis (7 stages for both humans and mice) and spermatogenesis (14 stages for humans and 20 stages for mice) [6–10]. We downloaded the raw data in FASTQ form and processed them with our unified pipeline for raising their comparability as described below.

Additionally, we added gene expression datasets (10 samples for human oogenesis, 14 samples for mouse oogenesis, and 411 samples for monkey oogenesis; 10,115 samples for human spermatogenesis, 29,552 samples for mouse spermatogenesis, and 19,476 samples for monkey spermatogenesis) from other data sources [16–19] as supplement and integrated them into Advanced Search.

### Processing of single-cell RNA-seq data

First, the reads of pooling library were separated into single cell by the barcodes as previously described [9]. The reads of each cell were filtered and trimmed with Trim Galore! (v0.6.6; https://www.bioinformatics.babraham.ac.uk/projects/trim_galore/) using default parameters. Next, the trimmed reads were aligned to hg38 (human) or mm10 (mouse) reference genome using STAR (v2.7.1a) [20] with default parameters. The ribosomal RNA (rRNA) reads were removed by RSeQC (v2.3.7) [21] with default parameters. Only uniquely mapped reads were retained, and the duplications were removed based on unique molecular identifier (UMI) information. The reads of exon model per million mapped reads (RPM) of each gene was calculated to normalize the gene expression data.

For the visualization of gene expression features, we used the Seurat (v4.0) package [22] to perform the RunTSNE function and the RunUMAP function (dims = 1:20) based on normalized RPM expression values. The marker genes of each developmental stage were identified by the FindAllMarkers function (min.pct = 0.25, logfc.threshold = 0.5, return.thresh = 0.01). Then we used the HOMER software [23] to perform the motif enrichment to find the TFs regulating the stage-specific marker genes with default parameters.

For the exhibition of gene expression in the Genome Browser, BAM files in the same developmental stage were merged and transferred into bigWig form with 50-bp windows using the bamCoverage function in deepTools (v3.4.3) [24].

### Processing of COOL-seq data and BS-seq data

The raw data of COOL-seq data and BS-seq were filtered and trimmed with Trim Galore! (v0.6.6; https://www.bioinformatics.babraham.ac.uk/projects/trim_galore/) and then aligned to hg38 (human) or mm10 (mouse) reference genome with Bismark (v0.22.3) [25] in single-end mode. The polymerase chain reaction (PCR) duplicates were removed by SAMtools [26]. We used the bismark_methylation_extractor function of Bismark (v0.22.3) [25] to calculate methylation level or chromatin accessibility of each covered cytosine. The number of “methylated” reads (reported as C) was divided by the total number of “methylated” and “unmethylated” reads (reported as C or T) at the same reference position. The average DNA methylation level was represented by the average level of WCG sites, and the chromatin accessibility was represented by the average level of GCH sites. The methylation level and chromatin accessibility in gene promoters were separately evaluated by the average levels of WCG sites and GCH sites within the regions from 1.5 kb upstream to 0.5 kb downstream of the transcription start site for each gene.

### Database interface

**Figure 1:**
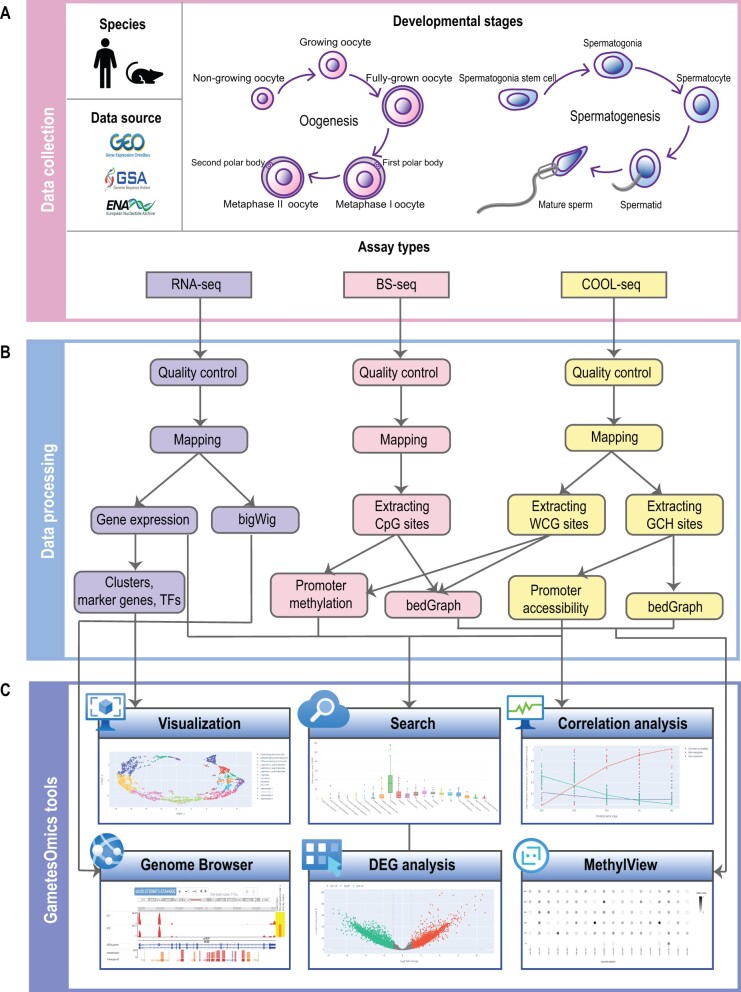
The framework of GametesOmics **A**. Data collection of multi-omics sequencing data. **B**. The custom pipeline to process the collected data for downstream applications and display in GametesOmics. **C**. The tools provided by GametesOmics. WCG sites includes ACG or TCG and GCH sites includes GCA, GCC or GCT. GEO, Gene Expression Omnibus; SRA, Sequence Read Archive; ENA, European Nucleotide Archive; RNA-seq, RNA sequencing; BS-seq, bisulfite sequencing; COOL-seq, chromatin overall omic-scale landscape sequencing; TF, transcription factor; DEG, differentially expressed gene.

We collected 66,267 samples (6689 samples for primary datasets and 59,578 samples for additional datasets) from different developmental stages in oogenesis (mainly divided into non-growing oocyte, growing oocyte, FGO, metaphase I oocyte, and metaphase II oocyte) and spermatogenesis (mainly divided into spermatogonia stem cell, spermatogonia, spermatocyte, spermatid, and mature sperm) [6–10,16–19] (**Figure 1**A; Tables S1 and S2). Then we processed the data with our unified pipeline and developed several tools for studying synergistic changes of different layers in gametogenesis (Figure 1B).

GametesOmics provides a user-friendly website as well as various useful functions including: (1) Search and Advanced Search for querying and displaying expression and epigenetic modification levels of interested genes; (2) Tools with Differentially expressed gene (DEG) analysis for applying differential analysis, Correlation analysis for simultaneously displaying the dynamic changes in gene expression and coordinated epigenetic alterations, Visualization for showing marker genes as well as master TFs, and MethylView for viewing the methylation in WCG sites or GCH sites; (3) Genome Browser and Ortholog for tracking and comparing expression and modifications of homologous genes between humans and mice (Figure 1C).

### Search and Advanced Search

The primary goal of Search and Advanced Search is to help users to retrieve the expression and epigenetic modification changes of their interested genes.

Users can input a gene (gene symbol, Entrez ID, or Ensembl ID) and then choose human or mouse in Search box on the home page. They will get quick access to the result page of their queried gene, which includes: the detailed descriptions [such as the gene official name, alias, functions, related Gene Ontology (GO) terms, and Reactome pathway(s)]; gene expression levels, promoter methylation levels, and promoter chromatin accessibility across the developmental stages in oogenesis or spermatogenesis.

Advanced Search supports users to input multiple genes at one time to access their expression or modifications in promoters. Users can also choose different datasets that they are interested in. This function may not only save the time when searching large batches of genes, but also display the correlation of these genes with various plot forms (such as line plot, bar plot, box plot, or heatmap).

### Tools

#### DEG analysis

DEGs between two developmental stages usually participate in driving the changes in the morphology or functions during the germ cell development. Therefore, identifying DEGs is important for screening the key genes which have the potential to determine the fate of oocytes or sperms.

By choosing the species, gamete types (oocyte or sperm), assay types, and developmental stages in the option page, the DEGs can be obtained by limma-voom method [27] between the selected stages. The result page will show the clusters of samples, heatmap of top 20 DEGs, and the DEG-enriched GO terms and Reactome pathways.

#### Correlation analysis

It has been revealed that the dynamic changes of epigenetic alterations including DNA methylation and chromatin accessibility can regulate gene expression in gametogenesis. Previous studies found that a number of gene promoters were accessible in human growing oocytes at an early stage to prepare for their transcription [9,28]. Therefore, the coordinate changes and correlation between the gene expression and epigenetic modifications may help us to delineate the regulatory mechanism in gametogenesis.

Correlation analysis provides a tool for users to view and compare the coordinate changes in expression levels, methylation levels, and chromatin accessibility of their interested genes across different developmental stages in gametogenesis. Researchers may be able to explore the relationship between the gene expression and epigenetic modifications, and to decode the molecular mechanism of the germ development and inheritance.

#### Visualization

Gametogenesis is a highly ordered and continuous process, which can be reflected and traced by the single-cell gene expression. Besides, by exploring the gene expression characteristics along the developmental trajectory, the biomarkers and key TFs of each stage could be screened.

Visualization is used to display gene expression features of each single cell. Users can select species, gamete types, and cluster methods in the option page. Then the result page will provide a visualization of the single-cell RNA-seq data. First, clustered plot exhibits the gene expression similarity and distance between these single cells. The developmental stages are marked with different colors, which may show the developmental trajectories of oogenesis or spermatogenesis. Second, users can obtain the heatmap of top 20 marker genes of any developmental stage. Third, upstream TFs regulating all the marker genes of this developmental stage would be displayed with a bubble plot. Using this module, users may be able to find the master TFs with high expression levels as well as critical factors and explore the regulatory mechanisms of the development process.

#### MethylView

The epigenetic modifications in different genomic regions can cause different regulatory effects on gene expression. For instance, it was revealed that hypermethylation in promoters or enhancers usually resulted in transcriptional silencing, while hypomethylation in gene bodies usually promoted gene expression [29]. Thus, accessing the epigenetic modifications across different genomic regions could help us discuss their regulation effects on transcription more specifically.

MethylView provides an access to the lollipop diagrams of each WCG site (reflecting DNA methylation level) or GCH site (reflecting chromatin accessibility) of selected species, gametes, and genomic regions. The color of each dot shows the level of DNA methylation or chromatin accessibility. Using this tool, users will directly view the epigenetic modification levels in any region such as intron, exon, and intergenic region, which would make the investigation of the epigenetic regulation more easily.

### Genome Browser and Ortholog

Genome Browser provides a window for users to directly view gene expression and epigenetic modification tracks of their interested developmental stages in a specific gene location or chromosome region. This function exhibits the transcription and modification on the reference genome in detail.

Mice are usually used as the mammalian experimental model in genetic studies for their similar genome with humans, easy manipulation on the genome, and short lifecycle [30]. Therefore, we can utilize the results of gene research in mice as the reference for human studies according to the corresponding genomic homologous regions. The Ortholog module is developed to view the expression and modification of any human–mouse homologous regions in the selected developmental stage. This function will not only help researchers to study the similarities as well as the divergences between humans and mice, but also promote the transformation from the experimental research into clinical applications.

## Applications

To present the utilization of GametesOmics, we used it for exploring the essential genes in the developmental stages of oogenesis and spermatogenesis. We applied the Visualization, Search, Advanced Search, DEG analysis, and Ortholog tools for the case studies.

### Screening the regulatory factors of spermatids in humans and mice

Spermatogenesis process mainly includes spermatogonia proliferation and differentiation, spermatocyte meiosis, and spermatid post-meiotic development [31]. The post-meiotic development, also called spermiogenesis, involves the formation of acrosome and flagella, condensation of the chromatin, elimination of extra cytoplasm, and transforming into spermatozoa [32,33]. This process is highly orchestrated and depends on the accurate regulation of many genes. However, the reports of the key factors of the spermatid post-meiotic development are limited.

**Figure 2:**
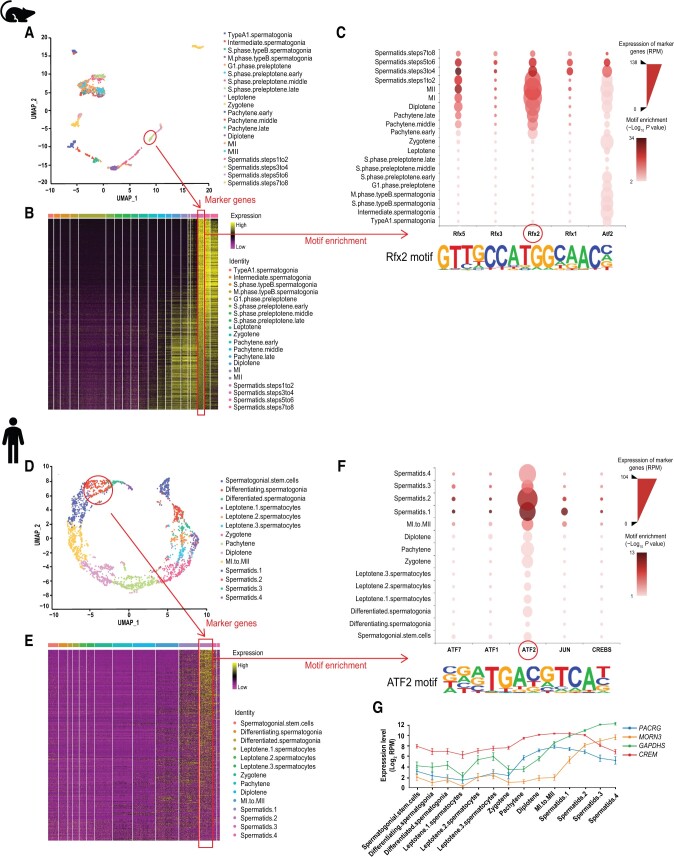
Screening the regulatory factors of spermatids in humans and mice **A**. UMAP plot showing the mouse spermatogenesis trajectory. Developmental stages are marked by different colors. “spermatids.steps3to4” stage is highlighted. **B**. Heatmap showing the expression of stage-specific genes of the “spermatids.steps3to4” stage in mouse spermatogenesis. **C**. Bubble plot showing the top 5 TFs enriched in the promoters of stage-specific genes of the “spermatids.steps3to4” stage, with selected Rfx2 motif displayed below. **D**. UMAP plot showing the human spermatogenesis trajectory. Developmental stages are marked by different colors. “spermatids.2” stage is highlighted. **E**. Heatmap showing the expression of stage-specific genes of the “spermatids.2” stage in human spermatogenesis. **F**. Bubble plot showing the top 5 TFs enriched in the promoters of stage-specific genes of the “spermatids.2” stage, with selected ATF2 motif displayed below. **G**. Line plot showing the expression of *PACRG*, *MORN3*, *GAPDHS*, and *CREM* during human spermatogenesis. UMAP, Uniform Manifold Approximation and Projection; RPM, reads of exon model per million mapped reads.

In mice, the single-cell spermatids displayed by the Uniform Manifold Approximation and Projection (UMAP) plot were at the end of the spermatogenesis trajectory (**Figure 2**A). Among the spermatids, the “spermatids.steps3to4” sub-stage was revealed to be quite essential as the single sphere acrosomal granule started to form, and the expression of genes related to chromatoid body formation, cilium movement, and acrosome formation also reached their peak levels [8,34]. We utilized the Visualization function in GametesOmics to investigate the master TFs of this developmental stage. The bubble plot showed that the promoters of stage-specific genes were enriched in Rfx2 motif, and *Rfx2* was highly expressed in spermatids (Figure 2B and C). Previous studies found that the spermiogenesis in male *Rfx2*^−/−^ mice was disrupted. Round spermatids failed to generate flagella and were not able to differentiate into elongated spermatids, along with altered expression of a large number of genes associated with the spermatogenesis, and eventually underwent apoptosis [35]. RNA-seq and ChIP-seq analyses have identified genes directly controlled by Rfx2 during spermiogenesis and found that they were involved in cilium function, cytoskeleton remodeling, and cell adhesion, which indicated that Rfx2 is an important TF for the round spermatid differentiation as well as flagellar biogenesis [36]. This verified the analytical result of the Visualization function.

In humans, the single-cell spermatids were also at the end of the spermatogenesis trajectory (Figure 2D). Among the spermatids, the “spermatids.2” sub-stage is important, in which the peanut agglutinin (a sperm-acrosome-specific marker) shapes as crescent and the transition nuclear proteins switch to protamine at this stage [8]. In the same way, we found the putative key TF in humans using the Visualization function. We found that the promoters of stage-specific genes were enriched in ATF2 motif, and *ATF2* was highly expressed and enriched in human spermatids (Figure 2E and F), which have not been studied in human spermatogenesis. ATF2 has been reported to play an important role during human early development. It can bind the promoters of many key genes regulating inflammatory signaling, cell cycle control, glycosylation and so on [37–39].

We downloaded the target genes of ATF2 from Cistrome database (http://cistrome.org/db/). The downstream includes several regulatory genes related to the development of the sperm, such as *PACRG*, *MORN3*, *GAPDHS*, and *CREM*. We input these downstream target genes into the Advanced Search tool; it turned out that they were highly expressed in the spermatids in humans (Figure 2G). *Pacrg* is found to be highly expressed in mouse testis and is required to build the sperm flagella [40]. In humans, loss of *PACRG* can cause male sterility [41], and null mutations in *PACRG* are observed in patients with severe sperm motility disorders [42]. *Morn3* is highly expressed in male germ cells of mice and is localized in the acrosome and manchette, which are also specific and essential structures for spermiogenesis [43]. *GAPDHS* is a sperm-specific expressed gene and plays an essential role in energy production for sperm motility and male fertility. It has been also studied as a potential male contraceptive target for humans [44]. *CREM* is reported to be important in the structuring of mature spermatozoa [45]. Lack of the *Crem* gene can cause mice becoming infertile and the arrest of the round spermatids. Besides, *CREM* has been confirmed to be expressed in human germ cells and might act as a switch for the human spermatogenesis [46]. In conclusion, it indicates that the screened ATF2 by GametesOmics might be a key regulator of the spermatid post-meiotic development in humans.

### Screening the key genes involved in the growth of oocytes in humans and mice

In mammals, the small and non-growing oocytes need weeks or months to grow into more than 100-fold of their volume [47]. In this process, they undergo a burst of transcription and translation to reach their full size, which is regarded as one of the foundations for fertilization and embryogenesis [48]. Growing oocyte I (GO1) is the main stage characterized by the rapid growth compared with FGO. Therefore, genes highly expressed in GO1 are essential for oogenesis in humans and mice. However, the cross-species conservation and diversity of gene expression as well as epigenetic modifications between humans and mice need to be further investigated.

**Figure 3:**
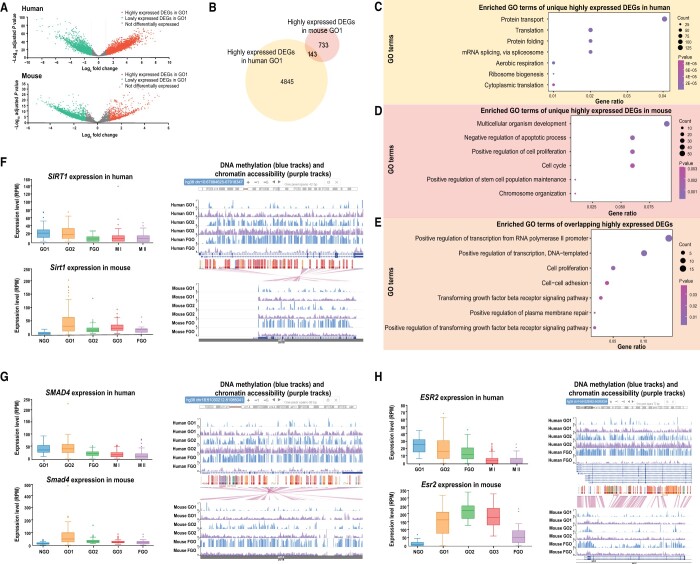
Screening the key genes involved in oocyte growth in humans and mice **A**. Volcano plot showing the fold changes and *P* values of DEGs between GO1 and FGO in humans and mice. **B**. Venn diagram of the highly expressed DEGs in humans and mice at the GO1 stage. **C**. GO enrichment of the unique highly expressed DEGs in human GO1 stage. **D**. GO enrichment of the unique highly expressed DEGs in mouse GO1 stage. **E**. GO enrichment of the overlapping highly expressed DEGs in human and mouse GO1 stages. **F**. The gene expression levels (showed by the box plot), the DNA methylation tracks (showed by UCSC Genome Browser in blue), and the chromatin accessibility tracks (showed by UCSC Genome Browser in purple) of *SIRT1/Sirt1* in human and mouse oogenesis. **G**. The gene expression levels (showed by the box plot), the DNA methylation tracks (showed by UCSC Genome Browser in blue), and the chromatin accessibility tracks (showed by UCSC Genome Browser in purple) of *SMAD4*/*Smad4* in human and mouse oogenesis. **H**. The gene expression levels (showed by the box plot), the DNA methylation tracks (showed by UCSC Genome Browser in blue), and the chromatin accessibility tracks (showed by UCSC Genome Browser in purple) of *ESR2*/*Esr2* in human and mouse oogenesis. GO1, growing oocyte I; GO2, growing oocyte II; GO3, growing oocyte III; M I, metaphase I oocyte, M II, metaphase II oocyte; NGO, non-growing oocytes; FGO, fully-grown oocyte; GO, Gene Ontology; UCSC, University of California Santa Cruz.

To explore the key regulatory genes involved in the growth of oocytes in humans and mice, we used DEG analysis in GametesOmics to obtain the DEGs between GO1 and FGO (**Figure 3**A). It turned out that 4845 genes in humans and 733 genes in mice were relatively highly expressed in GO1 (Figure 3B). GO analysis indicated that the unique highly expressed DEGs in humans were enriched in terms such as “protein transport”, “protein folding”, and “ribosome biogenesis”, while the unique highly expressed DEGs in mice were enriched in terms such as “multicellular organism development”, “negative regulation of apoptotic process”, and “chromosome organization” (Figure 3C and D). The 143 human and mouse overlapping highly expressed DEGs were enriched in “positive regulation of transcription”, “proliferation”, and “growth factor signaling”, implying that they might be conservatively expressed across species and function in the growing oocytes in humans and mice (Figure 3E).

In addition, we found that the overlapping highly expressed DEGs could be regulated by divergent molecular mechanisms. For example, Sirtuin 1 (SIRT1) is one of the family of nicotine adenine dinucleotide (NAD)^+^-dependent deacetylases and is associated with metabolic challenges, DNA repair, stress response, and reproductive aging [49,50]. It also participates in chromatin organization by remodeling chromatin structure and accessibility. In mice, the aging oocytes show defective expression of SIRT1 protein [51]. In humans, *SIRT1* has been proven to be related to the process of proliferation and activation of steroidogenesis [52]. *SIRT1/Sirt1* was highly expressed in the growing oocyte (Figure 3F). As shown in the result of Ortholog in GametesOmics, as for humans, the low DNA methylation and high chromatin accessibility levels of the *SIRT1* promoter might promote the expression of *SIRT1* at GO1 stage. However, as for mice, only the DNA methylation alterations were investigated to be associated with the regulation of *Sirt1* expression, while the chromatin accessibility showed relatively stable during the oogenesis (Figure 3F). SMAD4, which forms a complex with the phosphorylated R-SMADs, can regulate the genes controlling the cell cycle and cell fate determination [53]. SMAD4 also functions as the central molecule of the transforming growth factor-β (TGF-β) signaling pathway, which is associated with primordial follicle formation, ovulation, and signaling between the pituitary and ovary [54–56]. *SMAD4* was found to be specially expressed in oocytes and follicular somatic cells. *Smad4* conditional knockout mice were observed to have reduced antral follicles and ovulation rates, as well as defects in cumulus cells; their fertility decreased, and half were infertile by six months of age [57]. We found that *SMAD4* and *Smad4* were highly expressed in growing oocytes in humans and mice, respectively (Figure 3G), implying that *SMAD4* might play an essential role in the oocyte growth in humans as *Smad4* in mice. Like *SIRT1*, *SMAD4* promoter also showed relatively low DNA methylation and high chromatin accessibility at this stage in humans, while chromatin accessibility of *Smad4* was stable in mice (Figure 3G). Besides, estrogen receptor 2 (ESR2) is an essential estrogen receptor, and its polymorphisms and mutations are found to be associated with ovulatory dysfunctions in humans [58,59]. As for mice, loss of function of *Esr2* can lead to defective follicle development and ovulation [60]. *ESR2* and *Esr2* were highly expressed in human and mouse growing oocytes, respectively (Figure 3H), and showed similar modification alterations with *SMAD4/Smad4.*

In summary, the homologous DEGs identified by GametesOmics may play important roles in the growth of oocytes. Furthermore, the conservation and diversity in gene expression as well as epigenetic modifications across humans and mice during gametogenesis can be explored through GametesOmics.

Collectively, GametesOmics provides informative datasets and useful tools for biologists to screen master TFs and regulatory genes during gametogenesis.

## Discussion

To our knowledge, GametesOmics is the first database which specifically deposits multi-omics information of oogenesis and spermatogenesis in humans and mice. These single-cell sequencing data were processed with a unified pipeline, making it viable for the quantization and comparation between these datasets. It provides various useful functions for searching and viewing gene expression and epigenetic modifications, as well as tools for DEG identification, synergistic regulatory network investigation, and master factor screening during gametogenesis. Users can easily utilize GametesOmics to analyze these integrated sequencing data according to their research demand.

Previous studies have portrayed genetic and epigenetic landscapes in oogenesis as well as spermatogenesis, but the key factors driving the changes of morphology and function during these processes need to be further discovered. In our applications based on GametesOmics, we screened Rfx2 as a potential master TF in mouse spermatids. Studies demonstrated that deletion of *Rfx2* disrupted the differentiation into elongated spermatids [35,36], which proved the practicability and reliability of our database. Also, based on our developed tools, we identified *SIRT1/Sirt1*, *SMAD4/Smad4*, and* ESR2/Esr2* as key genes during human and mouse oocyte growth. *SIRT1* was reported to be associated with proliferation metabolic challenges, DNA repair, and reproductive aging in humans [52]. *Smad4* conditional knockout reduced antral follicles and ovulation rates in mice [57]. *ESR2* encodes an essential estrogen receptor and is reported to be associated with follicle development and ovulation [58–60]. This suggests the validity of the tools in GametesOmics. We also found the conserved expression and divergent epigenetic modifications of *SIRT1/Sirt1*, *SMAD4/Smad4*, and *ESR2/Esr2* in humans and mice, supporting the practicability of GametesOmics.

Taken together, we believe that GametesOmics will help researchers to dissect the molecular regulatory mechanisms in germ cell development from multi-layers and facilitate the exploration of genetic and epigenetic inheritance between generations. Besides, it will also be possible to provide clues in deciphering and curing reproductive disorders as well as genetic diseases in clinic.

In the future, GametesOmics will continuously update and integrate more genetic and epigenetic sequencing data of single cells across oogenesis and spermatogenesis from newly published research. Besides, we design to optimize and expand the functions according to the feedback of our users for offering more convenient applications as much as possible.

## Supplementary Material

qzad004_Supplementary_Data

## Data Availability

The GametesOmics database is available at http://gametesomics.cn/, and can be accessed without registration or login.

## CRediT author statement

**Jianting An:** Data curation, Methodology, Validation, Formal analysis, Investigation, Writing – original draft. **Jing Wang:** Data curation, Validation, Investigation. **Siming Kong:** Data curation, Resources. **Shi Song:** Resources, Investigation. **Wei Chen:** Resources, Investigation. **Peng Yuan:** Resources. **Qilong He:** Resources. **Yidong Chen:** Resources. **Ye Li:** Resources. **Yi Yang:** Resources. **Wei Wang:** Resources. **Rong Li:** Resources. **Liying Yan:** Conceptualization, Resources, Writing – review & editing, Supervision, Project administration. **Zhiqiang Yan:** Conceptualization, Resources, Methodology, Writing – review & editing, Supervision, Project administration. **Jie Qiao:** Conceptualization, Resources, Writing – review & editing, Supervision, Project administration, Funding acquisition. All authors have read and approved the final manuscript.
