# Supplementary material for: GametesOmics: A Comprehensive Multi-omics Database for Exploring the Gametogenesis in Humans and Mice
Source: Genomics Proteomics Bioinformatics. 2023 Dec 22;22(1):qzad004. doi: 10.1093/gpbjnl/qzad004 (PMC12012701; doi:10.1093/gpbjnl/qzad004)
Supplement: qzad004_Supplementary_Data [file qzad004_supplementary_data.zip › Supplemental_Table_S1-done.docx]

**Table S1 Statistics of the collected primary data in GametesOmics**

| **Gametes** | **Species** | **Data type** | **Developmental stages** | **Sub stages** | **Cell count** | **GEO accession No.** | **Ref. (PMID)** |
| --- | --- | --- | --- | --- | --- | --- | --- |
| Oocyte | Human | RNA-seq | Growing oocyte | GO1 | 40 | GSE154762 | 33957080 |
|  |  |  | Growing oocyte | GO2 | 46 |  |  |
|  |  |  | Fully-grown oocyte | FGO | 81 |  |  |
|  |  |  | Metaphase Ⅰ oocyte | MI | 155 |  |  |
|  |  |  | Metaphase Ⅱ oocyte | MII | 90 |  |  |
|  |  |  |  | **Sum** | 412 |  |  |
| Oocyte | Human | COOL-seq | Growing oocyte | GO1 | 40 | GSE154762 | 33957080 |
|  |  |  | Growing oocyte | GO2 | 46 |  |  |
|  |  |  | Fully-grown oocyte | FGO | 81 |  |  |
|  |  |  | Metaphase Ⅰ oocyte | MI | 155 |  |  |
|  |  |  | Metaphase Ⅱ oocyte | MII | 90 |  |  |
|  |  |  |  | **Sum** | 412 |  |  |
| Oocyte | Mouse | RNA-seq | Non-growing oocyte | NGO | 45 | GSE114822 | 30560925 |
|  |  |  | Growing oocyte | GO1 | 52 |  |  |
|  |  |  | Growing oocyte | GO2 | 44 |  |  |
|  |  |  | Growing oocyte | GO3 | 45 |  |  |
|  |  |  | Fully-grown oocyte | FGO | 36 |  |  |
|  |  |  |  | **Sum** | 222 |  |  |
| Oocyte | Mouse | COOL-seq | Non-growing oocyte | NGO | 160 | GSE114822 | 30560925 |
|  |  |  | Growing oocyte | GO1 | 148 |  |  |
|  |  |  | Growing oocyte | GO2 | 139 |  |  |
|  |  |  | Growing oocyte | GO3 | 125 |  |  |
|  |  |  | Fully-grown oocyte | FGO | 142 |  |  |
|  |  |  | Metaphase Ⅱ oocyte | MII | 20 |  |  |
|  |  |  |  | **Sum** | 734 |  |  |
| Sperm | Human | RNA-seq | Spermatogonia stem cell | Spermatogonial stem cells | 160 | GSE106487 | 30174296 |
|  |  |  | Spermatogonia | Differentiating spermatogonia | 135 |  |  |
|  |  |  | Spermatogonia | Differentiated spermatogonia | 59 |  |  |
|  |  |  | Spermatocyte | Leptotene.1 spermatocytes | 99 |  |  |
|  |  |  | Spermatocyte | Leptotene.2 spermatocytes | 104 |  |  |
|  |  |  | Spermatocyte | Leptotene.3 spermatocytes | 121 |  |  |
|  |  |  | Spermatocyte | Zygotene | 262 |  |  |
|  |  |  | Spermatocyte | Pachytene | 307 |  |  |
|  |  |  | Spermatocyte | Diplotene | 339 |  |  |
|  |  |  | Spermatocyte | MI to MII | 353 |  |  |
|  |  |  | Spermatid | Spermatids.1 | 313 |  |  |
|  |  |  | Spermatid | Spermatids.2 | 193 |  |  |
|  |  |  | Spermatid | Spermatids.3 | 55 |  |  |
|  |  |  | Spermatid | Spermatids.4 | 43 |  |  |
|  |  |  |  | **Sum** | 2543 |  |  |
|  |  |  |  |  |  |  |  |
| **Gametes** | **Species** | **Data type** | **Developmental stages** | **Sub stages** | **Cell number** | **GEO accession No.** | **Ref. (PMID)** |
| Sperm | Mouse | RNA-seq | Spermatogonia | TypeA1 spermatogonia | 42 | GSE107644 | 30061742 |
|  |  |  | Spermatogonia | Intermediate spermatogonia | 44 |  |  |
|  |  |  | Spermatogonia | S phase type B spermatogonia | 70 |  |  |
|  |  |  | Spermatogonia | M phase type B spermatogonia | 58 |  |  |
|  |  |  | Spermatocyte | G1 phase preleptotene | 204 |  |  |
|  |  |  | Spermatocyte | Early S phase preleptotene | 60 |  |  |
|  |  |  | Spermatocyte | Middle S phase preleptotene | 58 |  |  |
|  |  |  | Spermatocyte | Late S phase preleptotene | 58 |  |  |
|  |  |  | Spermatocyte | Leptotene | 54 |  |  |
|  |  |  | Spermatocyte | Zygotene | 67 |  |  |
|  |  |  | Spermatocyte | Early pachytene | 68 |  |  |
|  |  |  | Spermatocyte | Middle pachytene | 55 |  |  |
|  |  |  | Spermatocyte | Late pachytene | 44 |  |  |
|  |  |  | Spermatocyte | Diplotene | 64 |  |  |
|  |  |  | Spermatocyte | MI | 49 |  |  |
|  |  |  | Spermatocyte | MII | 16 |  |  |
|  |  |  | Spermatid | Spermatids.steps1to2 | 49 |  |  |
|  |  |  | Spermatid | Spermatids.steps3to4 | 44 |  |  |
|  |  |  | Spermatid | Spermatids.steps5to6 | 42 |  |  |
|  |  |  | Spermatid | Spermatids.steps7to8 | 58 |  |  |
|  |  |  |  | **Sum** | 1204 |  |  |
| Sperm | Mouse | BS-seq | Spermatogonia stem cell | Kit+ Germline stem cell | 4 | GSE49623 | 24835570 |
|  |  |  | Spermatocyte | Spermatocyte | 4 |  |  |
|  |  |  | Spermatid | Spermatid | 4 |  |  |
|  |  |  | Mature sperm | Mature sperm | 4 |  |  |
|  |  |  |  | **Sum** | 16 |  |  |
|  |  |  |  |  |  |  |  |

*Note*: RNA-seq, RNA sequencing; BS-seq, bisulfite sequencing; COOL-seq, chromatin overall omic-scale landscape sequencing; GO1, growing oocyte I; GO2, growing oocyte Ⅱ; GO3, growing oocyte Ⅲ; M Ⅰ, metaphase Ⅰ oocyte, M Ⅱ, metaphase Ⅱ oocyte; NGO, non-growing oocytes; FGO, fully-grown oocyte; GEO, Gene Expression Omnibus; PMID, PubMed Unique Identifier.
