# Supplementary material for: GametesOmics: A Comprehensive Multi-omics Database for Exploring the Gametogenesis in Humans and Mice
Source: Genomics Proteomics Bioinformatics. 2023 Dec 22;22(1):qzad004. doi: 10.1093/gpbjnl/qzad004 (PMC12012701; doi:10.1093/gpbjnl/qzad004)
Supplement: qzad004_Supplementary_Data [file qzad004_supplementary_data.zip › Supplemental_Table_S2-done.docx]

**Table S2 Statistics of the additional data added in Advanced Search of GametesOmics**

| **Gametes** | **Species** | **Data type** | **Developmental stages** | **Sample count** | **GEO accession No.** | **Ref. (PMID)** |
| --- | --- | --- | --- | --- | --- | --- |
| Oocyte | Human | RNA-seq | Germinal vesicle | 5 | GSE197578 | 36042231 |
|  |  |  | Metaphase Ⅱ oocyte | 5 |  |  |
|  |  |  | **Sum** | 10 |  |  |
| Oocyte | Mouse | RNA-seq | Germinal vesicle | 7 | GSE197578 | 36042231 |
|  |  |  | Metaphase Ⅱ oocyte | 7 |  |  |
|  |  |  | **Sum** | 14 |  |  |
| Oocyte | Monkey | RNA-seq | Primordial follicle oocyte | 175 | GSE130664 | 32004457 |
|  |  |  | Primary follicle oocyte | 106 |  |  |
|  |  |  | Secondary follicle oocyte | 59 |  |  |
|  |  |  | Antral follicle oocyte | 71 |  |  |
| ­ |  |  | **Sum** | 411 |  |  |
| Sperm | Human | RNA-seq | Spermatogonia | 2167 | GSE142585 | 32504559 |
|  |  |  | Spermatocyte | 2247 |  |  |
|  |  |  | Round spermatid | 3185 |  |  |
|  |  |  | Elongating spermatid | 2516 |  |  |
|  |  |  | **Sum** | 10,115 |  |  |
| Sperm | Mouse | RNA-seq | Spermatogonia | 4239 | GSE112393 | 30146481 |
|  |  |  | Spermatocyte | 8792 |  |  |
|  |  |  | Round spermatid | 9923 |  |  |
|  |  |  | Elongating spermatid | 6598 |  |  |
| ­ |  |  | **Sum** | 29,552 |  |  |
| Sperm | Monkey | RNA-seq | Spermatogonia | 1156 | GSE142585 | 32504559 |
|  |  |  | Spermatocyte | 3731 |  |  |
|  |  |  | Round spermatid | 2945 |  |  |
|  |  |  | Elongating spermatid | 11,644 |  |  |
|  |  |  | **Sum** | 19,476 |  |  |
|  |  |  |  |  |  |  |
